# Progress in Medicine

**Published:** 1900-03-31

**Authors:** 


### Progress in Medicine.

### DISEASES OF CHILDREN.

(*Continued from page* 416.)

**Summer Diarrhœa in Infants.**—This subject is discussed by various authorities in the *Therapeutic Gazette*.[6] Jacobi points out that prevention is best secured by special care of the general health in the hot months, and by the avoidance of all errors in hygiene and diet. Such measures as frequent bathing in pleasantly cold water, the use of light body clothing made of thin flannel, and of very light bed clothing, with frequent washing of the mouth, will aid in securing this object. He finds over feeding to be an extremely common cause. There is no one pathological condition present, but the profuse diarrhœal discharge may depend on acute intestinal catarrh, or follicular enteritis, or streptococcic and bacillary gastro-enteritis. His treatment is as follows: If the stomach is involved irrigation should be employed with a ¾ per cent. solution of common salt until the fluid comes away clear. The intestines should be cleared out with castor oil and calomel (without opium), and with enemata. Vomiting and severe diarrhœa indicate the entire withholding of food for some hours, and then the use of rice or barley water, or albumen water. The last is composed of the white of one egg, five ounces of barley water, one or two teaspoonfuls of whisky, and enough salt and sugar to improve the taste. A teaspoonful of this mixture can be given every five or ten minutes, and is ample for sustenance. Later subnitrate of bismuth and opium are useful. General collapse or extreme exhaustion demands stimulation by means of hot water rectal injections, with a little whisky added, or 1-100 to 1-50 gr. strychnine subcutaneously, or a grain of camphor dissolved in spirit. Abdominal pain is best relieved by warm applications and opiates. He also finds benefit from the use of intestinal antiseptics, such as creasote, salol, betanaphthol, &c. Griffith thinks that rectal injections are of value only if there is much mucous discharge from the bowel. Opium is indispensable in the later stages, and controls especially the catarrhal condition by lessening the discharges. Holt lays stress on the fact that the initial condition is an acute intoxication from the absorption of putrefactive products, and the secondary condition is a catarrhal inflammation of the intestine. The toxins formed damage the intestinal epithelium, and after absorption, other viscera, such as the kidneys. He recommends the stoppage of all food, and the rapid emptying of the stomach and bowel. The stomach should be thoroughly washed out with water at 110 deg. Fahr., and the large intestine irrigated with a normal saline solution, three or four quarts being introduced through a soft catheter or rectal irrigating tube. He also employs calomel, castor oil, and magnesia, although regarding them as less valuable than the irrigation. Fischer strongly supports this active treatment of the stomach and bowels. After the stomach has been thoroughly washed out no food is given for some hours, but seltzer, Apollinaris, or hot water is allowed in small quantities. On the first day calomel in one quarter grain doses is given every two or three hours until free purgation results, and this is followed on the succeeding day by two or three 1-drachm doses of castor oil. When severe tenesmus is present he finds benefit from painting the lower end of the rectum with a two per cent. solution of cocaine. Writing on the same subject, Chapin[7] says that opium is contraindicated until the bowels have been thoroughly emptied and when cerebral symptoms threaten. In cases, however, in which rapid peristalsis and profuse glandular secretion persist a few moderate doses of opium may be of great service, and even save life.

**The Treatment of Acute Intussusception.**—Dr. R. Koch[8] says that purgatives should be avoided, but large enemas of ice-cold water, lavage of the stomach, light, careful massage of the abdomen, and opium internally may be tried. If these measures fail the case should be transferred to the surgeon, and it is to be remembered that forty-eight hours may be regarded as the limit within which an operation may be successfully undertaken. He relates one case ending in recovery, in which the operation was performed forty-six hours after the onset. Mr. Stansfield Collier[9] has found treatment by rectal injection to be unsatisfactory, only one case out of five being saved by this measure. On the other hand, the treatment by abdominal section, in which extensive manipulation of the bowel may be required, is apt to be followed by collapse, high fever, and death from cardiac failure. He, therefore, advises the combined method, in which the rectal injection of water is first employed under a pressure of 18 inches, with the probable reduction of the greater part of the intussusception, and then laparotomy is performed, a small incision being made over the tumour left, which is easily and rapidly reduced.

**Congenital Hypertrophy of the Pylorus.**—A further case of this recently-described affection, with necropsy, is related by Dr. F. E. Batten.[10] The patient was born in good health, and at the age of five weeks began to vomit without any apparent cause. Changes in the diet produced no effect on the vomiting, and wasting and constipation supervened. When first seen at the age of 11 weeks the child was emaciated, with flaccid abdomen, a clean tongue, and subnormal temperature. The stomach was distended and showed marked peristaltic action, and a firm mass could be felt in the region of the pylorus. The adoption of nasal feeding led to the gradual cessation of the vomiting, and well-digested stools were passed. At the age of 11 months an attack of acute gastro enteritis and broncho-pneumonia proved fatal. The necropsy showed a hypertrophied condition of the stomach and pylorus. Commenting on this case the author remarks that the benefit derived from the nasal feeding would seem to support Dr. Thomson's view that there is a functional disorder of the nerves of the stomach and pylorus. Peristalsis started by the act of deglutition leads to the regurgitation of food, but when this peristalsis is not induced—as in nasal feeding—the stomach will retain and digest the food.

**Congenital Idiopathic Dilatation of the Colon.**—A case of this nature is recorded by Professor Griffith.[11] The patient was three years old, and had suffered from constipation and painful distension of the abdomen since he was five months old. He was a well-nourished child with an enormous abdomen, which measured 25 inches at the umbilicus, and was everywhere tympanitic. In spite of medical treatment, and finally the operation of right inguinal colotomy, the patient gradually sank. At the necropsy no constriction in the colon was found. Discussing these obscure cases, of which he has collected 24 recorded by various writers, Dr. Griffith points out that the leading symptoms are constipation and abdominal distension, the latter evidently from dilatation of the colon. Everything points to an inability of the colon to propel soft contents rather than to a blocking by hard masses. The prognosis is unfavourable, for 18 of the 24 cases are known to have terminated fatally, and only two are known to have recovered—one after the formation of an artificial anus. The necropsies show a striking similarity in the principal features of the pathological anatomy. Enormous dilatation of the colon, in whole or in part, is present, with thickening of its walls. In a few cases the rectum was distended, but the small intestine escapes. There may be ulceration of the mucous membrane of the colon, especially if severe diarrhœa has occurred. The treatment which naturally suggests itself, namely, massage, electricity, the use of purgatives and enemata, and the rectal tube to relieve distension, may give temporary relief, but does not seem to have any effect on the underlying condition. The formation of an artificial anus (Halsted) and the total excision of the colon (Treves) are measures successfully carried out, but so far only on one occasion.

**The Pharyngeal Tonsil.**—Too much operative treatment is, according to Mr. Arbuthnot Lane,[12] directed to this organ. The familiar "adenoids" are in plain language a swelling of the lymphatic tissue, secondary to an infection of the mucous membrane of the naso pharynx. This, again, is but one manifestation of the general condition from which children who have a deficient respiratory capacity suffer. His line of treatment is to brace up these patients by respiratory exercises, and by teaching them to breathe through the nose. In this manner one can remove the nasal catarrh and the irritation and hypertrophy of the lymphatic tissues in the pharynx, and increase the lumen of the naso-pharynx. By this line of treatment he affirms that he has avoided the necessity for operation in cases for which surgical measures had previously been advised by others.

**Geographical Tongue.**—Dr. Jacobi[13] discusses this condition in a clinical lecture. Other descriptive terms, such as "mapped tongue," or "psoriasis," or "aerated exfoliation" of the tongue have been applied to it, but the main point is that this harmless condition should not be mistaken for anything else and actively treated. The tongue is red in large and small spots, with the papillæ prominent. These spots are surrounded by branching and interlacing lines where the epithelium is thickened and raised. The condition is often congenital, is persistent, and does not affect the patient in any way.

6 Aug., 1899; and Edin. Med. Jour., Oct., 1899. 7 Jour. Amer. Med. Assoc., July 22, 1899. 8 Der Kinderärzt, 1899, x., 49; and Treatment, Sept. 28, 189 . 9 The Lancet, Aug. 26, 1899. 10 The Lancet, Dec. 2, 1899. 11 Amer. Jou Med. Sci., Sept., 1899. 12 Edin. Med. Jour., Sept, 1899. 13 Arch. of Pediat, Jan., 1900.

